# Ultrasound Presentation of a Disseminated Fetal and Neonatal Rhabdoid Tumor

**DOI:** 10.1155/2018/6073204

**Published:** 2018-01-31

**Authors:** Yolaine Joueidi, Aline Rousselin, Céline Rozel, Philippe Loget, Dominique Ranchere Vince, Sylvie Odent, Franck Bourdeaut, Vincent Lavoue, Maela Le Lous

**Affiliations:** ^1^Department of Obstetrics and Gynaecology, CHU de Rennes, Hospital Anne de Bretagne, 16 Bd de Bulgarie, BP 90347, 35 203 Rennes Cedex 2, France; ^2^Department of Radiology, CHU de Rennes, Hospital Anne de Bretagne, 16 Bd de Bulgarie, BP 90347, 35 203 Rennes Cedex 2, France; ^3^Department of Pathology, CHU de Rennes, Hospital Pontchaillou, 2 rue Henri Le Guilloux, 35033 Rennes Cedex 9, France; ^4^Department of Pathology, Leon Berard Center, 28 rue Laennec, 69008 Lyon, France; ^5^Department of Genetics, CHU de Rennes, Hospital Anne de Bretagne, 16 Bd de Bulgarie, BP 90347, 35 203 Rennes Cedex 2, France; ^6^Medical School, University of Rennes 1, 2 Avenue du Professeur Léon Bernard, 35000 Rennes, France; ^7^Institut Curie, Paris Sciences et Lettres Research University, Inserm U830, Laboratoire de Genetique et Biologie des Cancers, 26 rue d'Ulm, 75005 Paris, France

## Abstract

This is a case report of a disseminated fetal rhabdoid tumor discovered at 32 weeks of gestation in a 29-year-old woman on immunosuppressive therapy. The mother consulted for a decrease in fetal movement. Fetal ultrasound showed signs of a disseminated tumor affecting the left armpit, liver, spleen, and limbs. A caesarian section was performed because of signs of fetal distress. Immunohistochemical analysis of a fetal biopsy showed deletion of the* SMARCB1* gene. Pathological analysis of the placenta showed a rhabdoid tumor invading both fetal and maternal compartments. The mother underwent a whole-body MRI, and no metastasis was found. To the best of our knowledge, this is the first report of a disseminated rhabdoid tumor invading both fetal and maternal compartments.

## 1. Case Report

**Figure 1:**
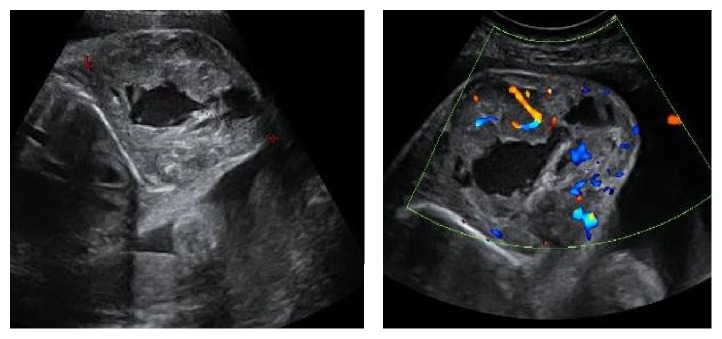
Fetal ultrasound showing the tumor in the left armpit.

**Figure 2:**
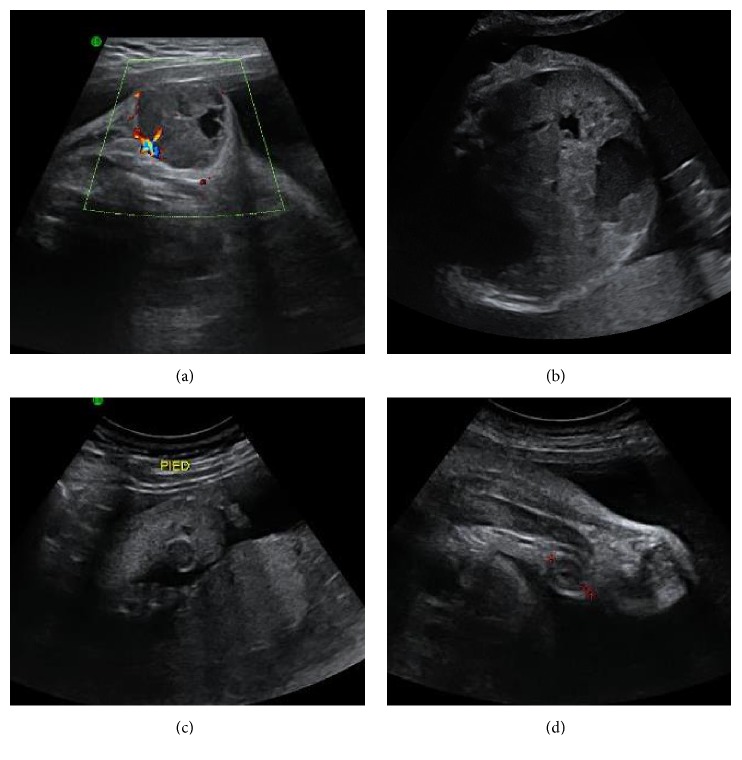
Other tumor sites on the iliac bone (a), intra-abdominal organs (b), left foot (c), and left thigh (d) found by ultrasound examination.

A 29-year-old pregnant woman who was being treated with azathioprine for autoimmune hepatitis consulted at 32 weeks of gestation for decreased fetal movement. Obstetric ultrasound revealed many heterogeneous, hypervascular fetal tumors in the left armpit (Figure 1), intra-abdominal organs, and subcutaneous space (Figure 2). There was no sign of fetal cardiac insufficiency or anemia (no pericardial effusion and the middle cerebral artery peak systolic velocity was 50 cm/sec or 1,07 MoM).

Fetal wellbeing was monitored daily and fetal lung maturity was induced at 32 weeks of gestation and 2 days with 12 mg of betamethasone repeated at 24 hours.

**Figure 3:**
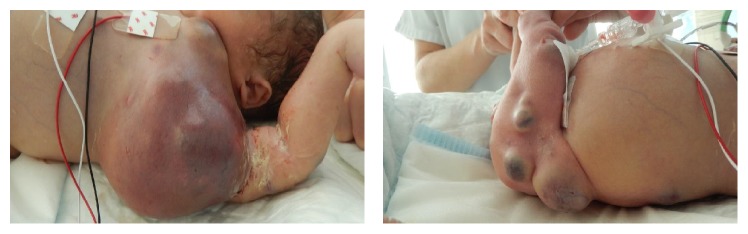
Pediatric examination of the newborn.

**Figure 4:**
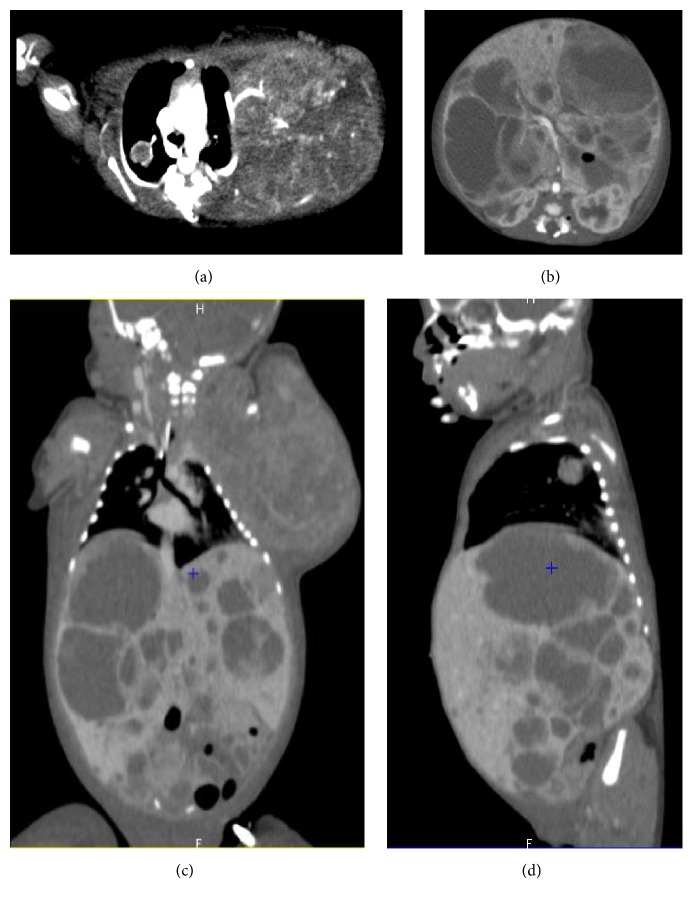
CT scan with a contrast agent showing the axillary mass compressing the chest (a) and numerous intra-abdominal tumors involving the liver, spleen, and right kidney (b, c, d) in axial, frontal, and sagittal views.

**Figure 5:**
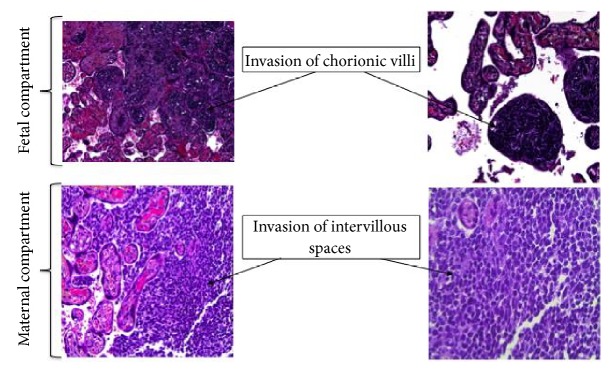
Pathological analysis of the placenta.

Two days later, a caesarian section was performed because of signs of fetal distress by fetal heart rate monitoring. The child presented immediately difficulties in adapting to extrauterine life and was cared for in the pediatric intensive care unit (Figure 3). First aid revealed that the child was anemic at birth (hemoglobin at 11,8 g/dl) with a pulmonary arterial hypertension on cardiac ultrasound. A body scan (Figure 4) and a biopsy of a cutaneous tumor were performed and showed characteristics of a rhabdoid tumor (small round nuclei, granular chromatin, and eosinophilic cytoplasm). The immunohistochemical analysis revealed a loss of* SMARCB1* protein expression and pathological examination of the placenta showed an invasion of both fetal and maternal compartments (Figure 5). Because of prematurity, circulatory failure, and severe renal dysfunction with anuria, chemotherapy was contraindicated, and the decision was made with the parents to withdraw care. The child died at day 5 of multivisceral failure. The mother underwent a whole-body MRI to identify potential metastasis, but none was found.

The* INI1/SMARCB1* analysis conducted by Sanger sequencing of all exons and exon/intron boundaries and a Multiplex Ligation-dependent Probe Amplification (MLPA) to detect copy number alterations revealed a homozygous deletion of the 9 exons in tumor DNA. This alteration was not found in the child's germ line nor in the parents. Therefore, the hypothesis of an inherited predisposition involving the* SMARCB1* gene was excluded, and the hypothesis of a postzygotic (de novo) genetic alteration was retained.

## 2. Discussion

Fetal rhabdoid tumors are rare, aggressive, and often diagnosed at metastatic stage. They have a poor prognosis with a high mortality rate before one year of life. The average age at diagnosis is 11 months. These tumors are reported most frequently in the central nervous system, kidneys, and soft tissue. They are sometimes discovered in utero following severe anemia secondary to tumor rupture or on the presence of many disseminated nodules [1]. Only 6 other cases of prenatal diagnosis of fetal rhabdoid tumor are described in literature. Their localization, the ultrasound description, the gestational age at diagnosis, and the placental tumor invasion are reported in Table 1 [2–8]. These tumors affected both boys and girls. They were in all cases diagnosed during the third trimester and invaded the placenta in 3 cases. They were most often metastatic at time of diagnosis and the birth occurred shortly after the diagnosis in all the cases with a survival less than 15 days.

The pathological diagnosis of rhabdoid tumor is based on characteristic cells with a round vesicular nucleus, a prominent nucleolus, and round or oval eosinophilic inclusion bodies. These cells show a deletion in chromosome 22q11 or mutations of the* SMARCB1* gene, a tumor suppressor gene, by immunohistochemical analysis. The majority of rhabdoid tumors arise from somatic loss of both copies of* SMARCB1*, which affects the formation of chromatin remodeling complexes.* SMARCB1* mutations can arise de novo or be secondary to a germline mutation, which should prompt genetic counseling [9]. In the 6 cases described in literature, 3 gave the genetic characteristics of the tumor but only one evoked an abnormality of chromosome 22q11.

Invasion of both fetal and maternal compartments of the placenta by tumor cells is extremely rare. In the literature, 36 cases of invasion of chorionic villi (fetal space) have been reported, but only 3 cases have been reported with invasion of the intervillous space with a risk of maternal dissemination of the tumor [10, 11] (Table 2). There have been no cases of maternal disease after complete examination, but this possibility should be explored in order to treat the mother as soon as possible, if necessary.

Azathioprine is a reported mutagen in vitro and in vivo in mice, but no case of human fetal malignancy has been reported in connection with maternal treatment [12].

Rhabdoid fetal tumors are aggressive with a difficult ultrasound diagnosis and a poor prognosis. The placenta should be analyzed for infiltration of the maternal side and tumor dissemination. Genetic counseling should be recommended for antenatal diagnosis of* SMARCB1* mutation, and preimplantation screening may be discussed in cases of germline mutation.

## Figures and Tables

**Table 1:** Cases of prenatal diagnosis of rhabdoid tumor.

Author	Year	Localization	Ultrasound exam	Newborn sex	WG at diagnosis	WG at Birth	Genetics	Fetal part of the Placenta involved	Maternal Part of the placenta involved	Child survival
White et al.	1999	Case 1: face and neck, anterior cranial fossa, metastasis	NS	F	33	NS	abnormality of chr 22q11	Yes	NS	Few minutes

Ohyama et al.	2000	Liver and skin metastasis	NS	M	33	33	NS	Yes	Yes	4 days

Staehelin et al.	2000	Right shoulder and sacral tumor	Cystic and solid tumor + polyhydramnios	F	31	32	46 XX, inv (11) (p13p15)	No	No	6 days
Hösli et al.	2001									

Leader et al.	2002	Neck and chest and left arm + metastasis	Vascularized tumor + polyhydramnios	F	29	30	47XX, +7[9]/47	NS	NS	5 days

Fuchs et al.	2004	Left kidney	Homogeneous vascularized mass of left kidney + polyhydramnios	M	26	29	NS	Analysis refused by parents	Analysis refused by parents	4 hours

Kwon et al.	2009	Right arm	Solid tumor + polyhydramnios	M	35	35	NS	No	No	12 days

Joueidi et al.	2014	Left armpit + metastasis	Vascularized heterogeneous tumor + polyhydramnios	M	32	32	Mutation SMARCB1 del 22q11	Yes	Yes	5 days

F = female; M = male; WG: weeks of gestation; Chr: chromosome; NS: not specified.

**Table 2:** Reported cases of placenta involvement by fetal malignancies.

Author	Fetal malignancy	Weight live	Survival	Fetal part of the placenta involved	Maternal part * *of the placenta involved	Kind of placenta involvement	Maternal disease Maternal follow up
Ohyama et al. (2000)	Epithelial tumor of the liver with metastasis	1774 g	4 days	Yes	Yes	Chorionic villi and intervillous spaces	No maternal disease

De Tar and Biggerstaff (2006)	Renal rhabdoid tumor with metastasis	3130 g	Died during neonatal care	Yes	Yes	Chorionic villi and intervillous spaces	No maternal disease 2 years

Reif et al. (2014)	Soft tissue sarcoma of the left index finger	3000 g	4 months	Yes	Yes	Chorionic villi and intervillous spaces	No maternal disease 9 months

Joueidi et al. (2014)	Disseminated rhabdoid tumor	2645 g	5 days	Yes	Yes	Chorionic villi and intervillous spaces	No maternal disease 2 years
